# Supplementary material for: Optimizing Vancomycin Therapy in Critically Ill Children: A Population Pharmacokinetics Study to Inform Vancomycin Area under the Curve Estimation Using Novel Biomarkers
Source: Pharmaceutics. 2023 Apr 25;15(5):1336. doi: 10.3390/pharmaceutics15051336 (PMC10220925; doi:10.3390/pharmaceutics15051336)
Supplement: Supplementary file 1 [file pharmaceutics-15-01336-s001.zip › pharmaceutics-2349307-supplementary.pdf]

Table S1. Goal PK sampling times relative to end of the vancomycin infusion.

**Dosing interval**	**<15 min**	**30 +/- 10 min**	**60 +/-15 min**	**180 +/- 30 min**	**360 +/- 60 min**	**720 +/- 60 min**	**<30 min prior to next dose**
**Q6H**	X	X	X	X			X
**Q8H**	X	X	X	X			X
**Q12H**	X	X		X	X		X
**Q18H**	X	X		X	X		X
**Q24H**	X		X		X	X	X

Table S2. Model training steps.

Model	Covariates	-2*LL (Δ)	AIC (Δ)
Step 1. Base model structure			
1-compartment	(WT/27)**0.75 on CL, (WT/27) on V_d	873.4	879.6
**2-compartment**	**(WT/27)**0.75 on CL and Q, (WT/27) on V1 and V2**	**812.3**	**820.5**
Step 2. 2-compartment model + renal function covariates on CL			
$eGFR_{Schwartz}$	(GFR/156)**TH1 on CL	797.2 (-15.1)	807.6 (-12.9)
**$eGFR_{Hoek}$**	**(HOEK/134)**TH1 on CL**	**782.9 (-29.4)**	**793.3 (-27.2)**
$eGFR_{FAS}$	(FAS/144)**TH1) on CL	791.1 (-21.2)	801.5 (-19.0)
Serum creatinine	(0.4/SCR)**TH1 on CL	797.2 (-15.1)	807.6 (-12.9)
Plasma cystatin c	(0.6/PCYSC)**TH1 on CL	782.5 (-29.8)	793.0 (-27.5)
Plasma NGAL	(95/PNGAL)**TH1 on CL	796.9 (-15.4)	807.3 (-13.2)
Step 3. 2-compartment model with $eGFR_{Hoek}$ on CL + other covariates			
ARC on CL	(CLARC**ARC) on CL; ARC=1 if $eGFR_{Hoek}$ > 130 ml/min/1.73 m^2	777.2 (-5.7)	789.8 (-3.5)
Age on CL	((AGE/10)**TH2) on CL	778.8 (-4.1)	791.4 (-1.9)
Age as Hill function on CL	(AGE**HILL/((AGE50**HILL)+(AGE**HILL))) on CL	777.9 (-5.0)	792.7 (-0.6)
Age on V1	((AGE/10)**TH2) on V1	778.0 (-4.9)	790.6 (-2.7)
Female sex on CL	(TH2**FEM); FEM=1 if female, 0 if male	776.7 (-6.2)	789.3 (-4.0)
Female sex on V1	(TH2**FEM); FEM=1 if female, 0 if male	780.6 (-2.3)	793.1 (-0.2)
Vasopressor receipt on CL	TH2**VASO on CL; VASO=1 if active vasopressor receipt	774.8 (-8.1)	787.4 (-5.9)
Vasopressor receipt on V1	TH2**VASO on V1; VASO=1 if active vasopressor receipt	779.3 (-3.6)	791.9 (-1.4)

PIM3 score on CL	(PIM3/2.82)**TH2 on CL	779.1 (-3.8)	791.7 (-1.6)
PIM3 score on V1	(PIM3/2.82)**TH2 on V1	777.7 (-5.2)	790.3 (-3.0)
SCr on CL	(0.4/SCR)**TH1 on CL	777.1 (-5.8)	789.7 (-3.6)
Plasma NGAL on CL	(95/PNGAL)**TH1 on CL	777.5 (-5.4)	790.1 (-3.2)
Urinary NGAL on CL	(50.1/UNGAL)**TH2 on CL	774.3 (-8.6)	786.9 (-6.4)
**Log urinary NGAL on CL**	**TH2**LUNGAL on CL; LUNGAL is the natural logarithm of uNGAL/UCr**	**771.3 (-11.6)**	**783.9 (-9.4)**
Urinary KIM-1 on CL	(3.3/UKIM1)**TH2 on CL	778.3 (-4.6)	790.9 (-2.4)
Log urinary KIM-1 on CL	TH2**LUKIM1 on CL; LUKIM1 is the natural logarithm of uKIM-1/UCr	775.1 (-7.8)	787.7 (-5.6)
Urinary OPN on CL	(4900/UOPN)**TH2 on CL	779.6 (-3.3)	792.2 (-1.1)
Log urinary OPN on CL	TH2**LUOPN on CL; LUOPN is the natural logarithm of uOPN/UCr	774.1 (-8.8)	786.7 (-6.6)
Urinary CysC on CL	(84/UCYSC)**TH2	778.2 (-4.7)	790.8 (-2.5)
Log urinary CysC on CL	TH2**LUCYSC on CL; LUCYSC is the natural logarithm of uCysC/UCr	773.9 (-9.0)	786.4 (-6.9)

Abbreviations: CysC, cystatin c; EGFR, estimated glomerular filtration rate; KIM-1, kidney injury molecule-1; NGAL, neutrophil gelatinase-associated lipocalin; OPN, osteopontin; PIM3, Pediatric Index of Mortality 3; SCr, serum creatinine; V1, central volume; V2, peripheral volume; V_d, volume of distribution; WT, weight.

Table S3. Biomarker concentrations at time of PK sampling.

Biomarker	Model training group (n = 30)	Model testing group (n = 20)	Wilcoxon rank sum p-value
Serum creatinine, mg/dL	0.30 (0.20–0.48)	0.35 (0.19–0.50)	0.92
Plasma CysC, mg/L	0.55 (0.4–0.7)	0.6 (0.5–0.8)	0.64
Plasma NGAL, ng/mL	83.6 (56.0–178.2)	102 (74.3–139.2)	0.85
Urine Cysc, ng/mg creatinine	103.5 (66.5–186.0)	121.0 (70.7–211.2)	0.69
Urine KIM-1, ng/mg creatinine	3.0 (1.6–7.6)	3.7 (1.4–8.0)	0.97
Urine NGAL, ng/mg creatinine	60.3 (24.1–249.8)	35.3 (18.7–62.1)	0.27
Urine osteopontin, ng/mg creatinine	4680.8 (2657.2–9449.5)	3387.3 (1949.1–5614.2)	0.35
Results reported as median (IQR).			

Table S4. Population PK parameter estimates for the full model, $EGFR_{Hoek}$ model, and $EGFR_{Schwartz}$.

	**Full model**	**$eGFR_{Hoek}$**	**$eGFR_{Schwartz}$**
	**Weighted parameter estimate, median (95th percentile)**	**Weighted parameter estimate, median (95th percentile)**	**Weighted parameter estimate, median (95th percentile)**
**CL0**	3.31 (2.53–4.22)	2.61 (2.10–3.05)	2.82 (2.56–3.23)
**CL_{EGFR}**	0.85 (0.22–0.90)	1.00 (0.84–1.13)	0.32 (0.07–0.76)
**CL_{NGAL}**	0.94 (0.96–1.00)	N/A	N/A
**V_C0**	3.50 (2.72–7.09)	5.09 (3.23–6.25)	5.09 (3.42–5.74)
**Q0**	7.09 (4.76–7.97)	6.18 (4.47–7.76)	6.27 (4.49–7.43)
**V_P0**	7.75 (6.63–13.80)	8.30 (7.04–12.61)	7.33 (6.52–8.62)

Models parameterized as:

$CL = CL0 \cdot (WT/27)^{0.75} \cdot (eGFR/134)^{CLEGFR} \cdot (CL_{NGAL})^{LNGAL}$, for Full model[a].

$CL = CL0 \cdot (WT/27)^{0.75} \cdot (eGFR/134)^{CLEGFR}$, for $eGFR_{Hoek}$ model[a].

$CL = CL0 \cdot (WT/27)^{0.75} \cdot (eGFR/156)^{CLEGFR}$, for $eGFR_{Schwartz}$ model[b].

$V1 = V_C0 \cdot (WT/27)^1$.

$Q = Q0 \cdot (WT/27)^{0.75}$.

$V2 = V_P0 \cdot (WT/27)^1$.

LNGAL is the natural logarithm of the urinary NGAL concentration normalized to urinary creatinine (uNGAL/uCr).

[a] eGFR calculated using plasma cystatin c and Hoek equation.

[b] eGFR calculated using plasma creatinine and bedside Schwartz equation.

Table S5. Summary of optimal sampling times relative to the end of the vancomycin infusion among model testing group.

	**Full model**	**eGFR$_{Hoek}$ model**	**eGFR$_{Schwartz}$ model**
Single optimally timed sample			
Mean (standard deviation)	2.2 (1.7)	2.3 (1.7)	2.9 (1.2)
Median (range)	2 (0–5.4)	2.75 (0–4.75)	3.1 (0.4–4.75)
Two optimally timed samples, first sample			
Mean (standard deviation)	0.875 (1.2)	1.2 (1.2)	2.25 (1.3)
Median (range)	0.25 (0–3.25)	0.5 (0–3.5)	2.75 (0–4.75)
Two optimally timed samples, second sample			
Mean (standard deviation)	3.4 (1.7)	3.4 (1.0)	4.2 (0.6)
Median (range)	3.6 (0.75–7)	3.25 (1–5.75)	4.25 (3.25–5.0)
All times reported in hours relative to the end of the vancomycin infusion.			

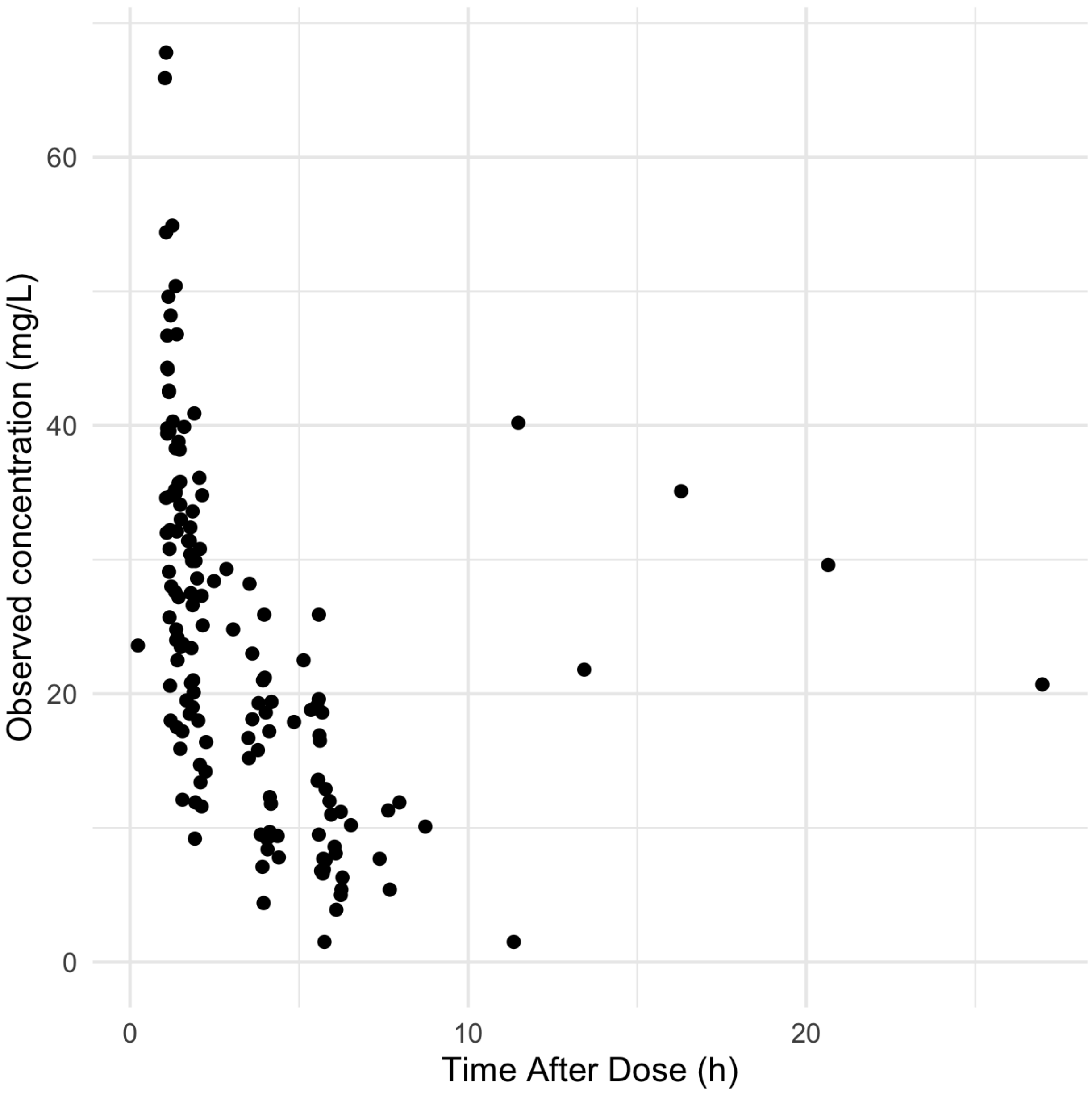

Figure S1. Observed concentrations versus time after dose for the model testing group.

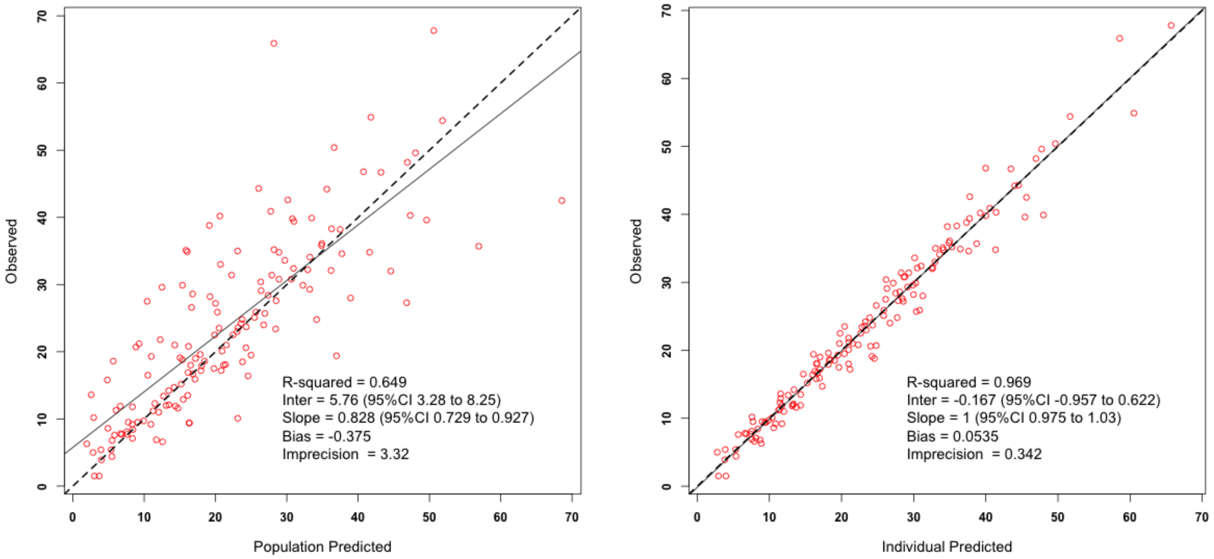

Figure S2. Observed versus population and individual predicted concentration plots of the full model.
